# Supplementary material for: Cell-Extrinsic Differentiation Block Mediated by EphA3 in Pre-Leukaemic Thymus Contributes to Disease Progression
Source: Cancers (Basel). 2021 Jul 31;13(15):3858. doi: 10.3390/cancers13153858 (PMC8345401; doi:10.3390/cancers13153858)
Supplement: Supplementary file 1 [file cancers-13-03858-s001.zip › cancers-1261331-supplementary.pdf]

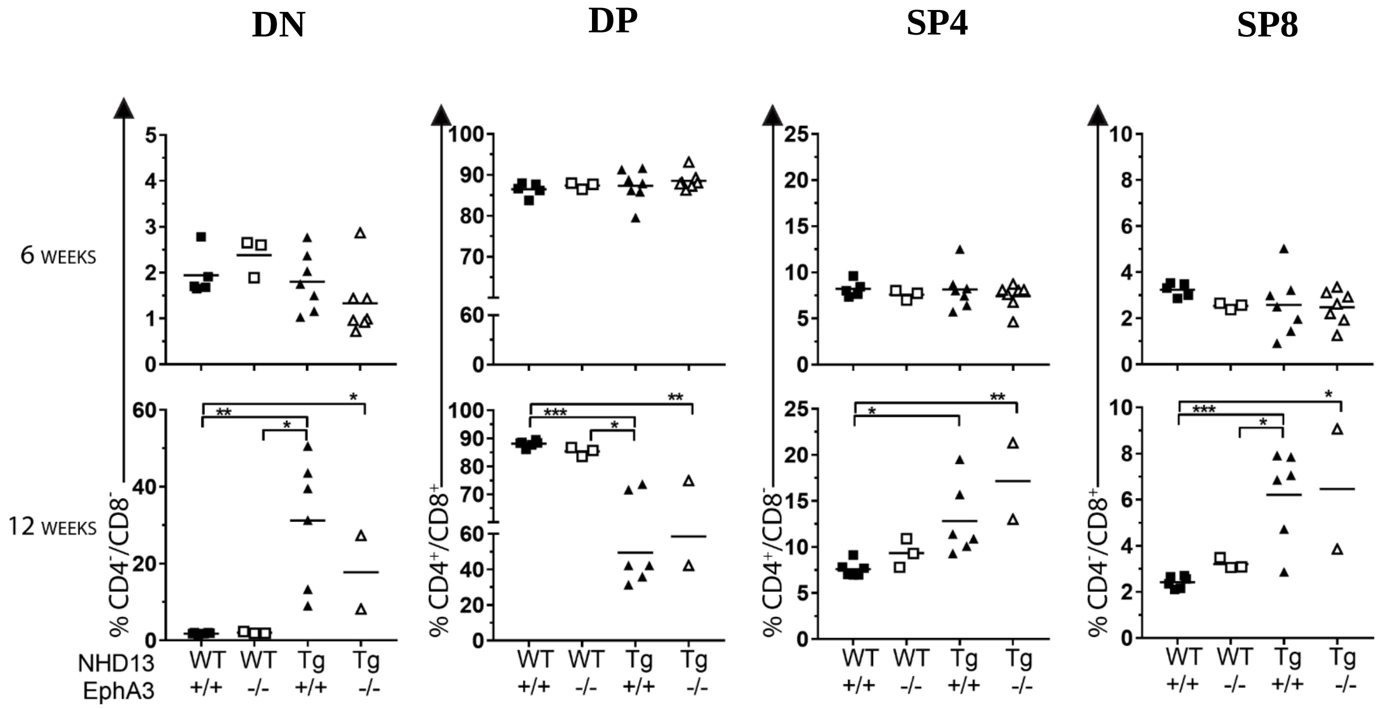

Figure S1. Quantitation of Double Negative (DN), Double Positive (DP), Single Positive $CD4^+$ (SP4) and Single Positive $CD8^+$ (SP8) populations

Quantitation of DN ($CD4^-/CD8^-$), DP ($CD4^+/CD8^+$), SP4 ($CD4^+/CD8^-$) and SP8 ($CD4^-/CD8^+$) in 6 and 12 week old mice of each indicated gentoype. Data represent the mean and points represent individual mice 3-8 per group. *P-values* were calculated using Student's T-test (*<0.05, **<0.005, ***<0.0005).

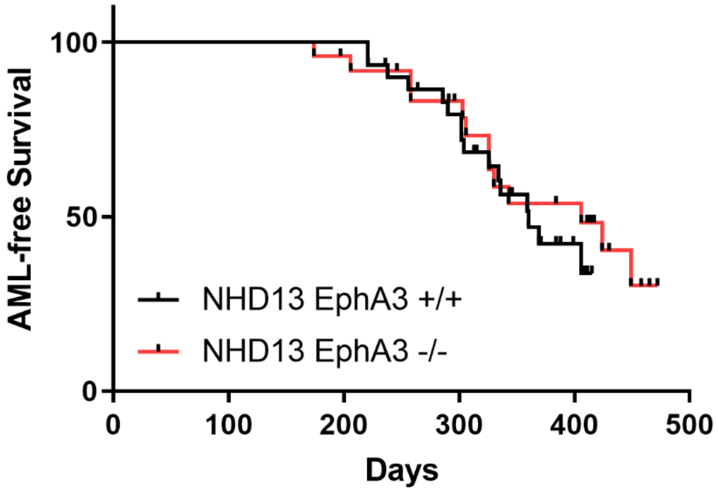

Figure S2. EphA3 deletion does not impact the incidence of AML in the NHD13 mice. Kaplan-Meier plot of T-ALL-free survival for each indicated genotype, with deaths due to causes other than AML censored.

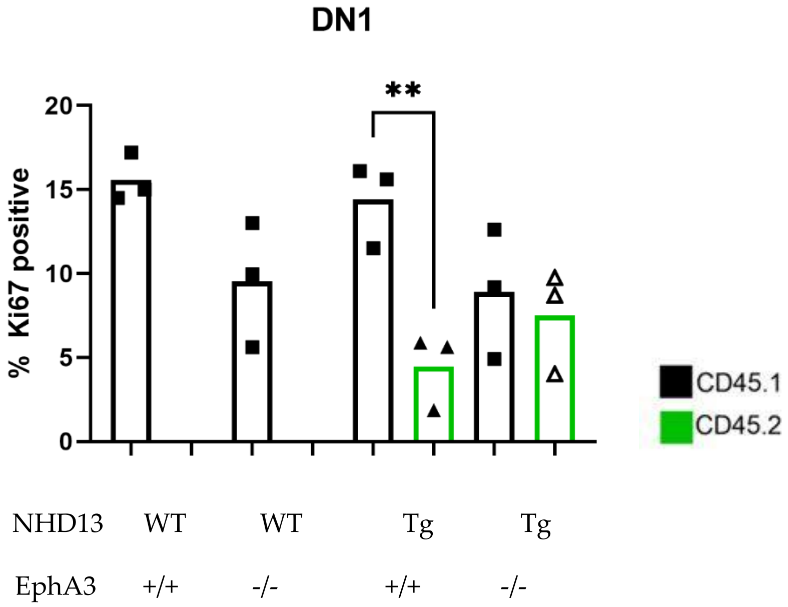
DN1
**
% Ki67 positive
20
15
10
5
0
CD45.1
CD45.2
NHD13 WT WT Tg Tg
EphA3 +/+ -/- +/+ -/-

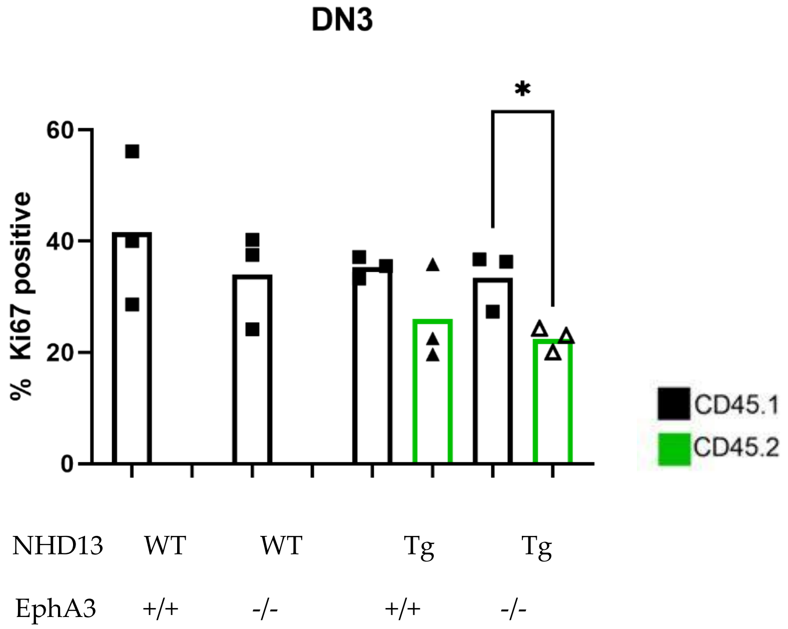
DN3
*
% Ki67 positive
60
40
20
0
CD45.1
CD45.2
NHD13 WT WT Tg Tg
EphA3 +/+ -/- +/+ -/-

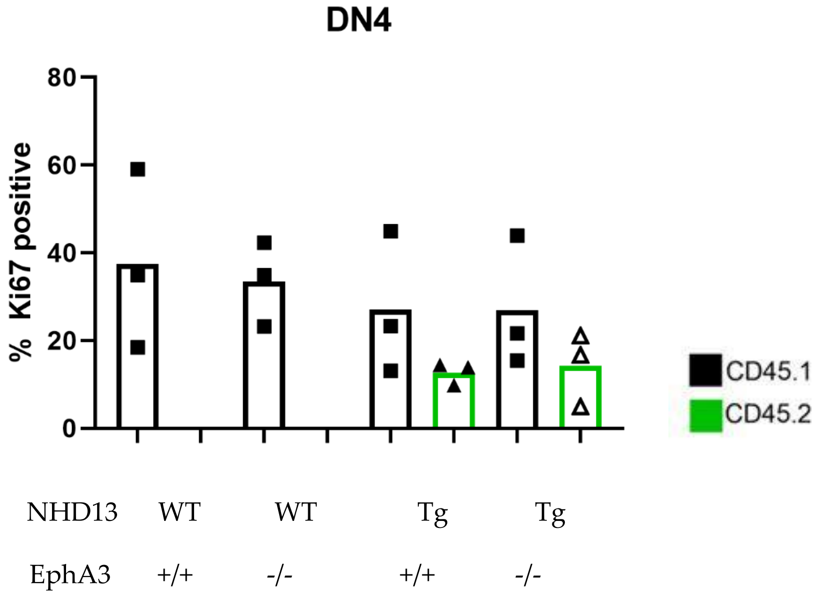

Figure S3. EphA3 deletion does not impact the cell cycle of wild type recipient cells in DN1, DN3 or DN4. Cell division analysis of DN1, DN3 and DN4 thymocytes in the primary transplant recipient. Recipient (CD45.1) or donor (CD45.2) thymocytes were analysed for cell division/ quiescent status using Ki67 and DNA (Hoechst) stains. Bars represent the mean, points represent individual mice, n = 3.
